# Comparison of beta peak detection algorithms for data-driven deep brain stimulation programming strategies in Parkinson’s disease

**DOI:** 10.1038/s41531-024-00762-7

**Published:** 2024-08-09

**Authors:** Sunderland K. Baker, Erin M. Radcliffe, Daniel R. Kramer, Steven Ojemann, Michelle Case, Caleb Zarns, Abbey Holt-Becker, Robert S. Raike, Alexander J. Baumgartner, Drew S. Kern, John A. Thompson

**Affiliations:** 1 Pennsylvania State University, Department of Biobehavioral Health, University Park, PA 16802 USA; 2 University of Colorado Anschutz Medical Campus, Department of Neurosurgery, Aurora, CO 80045 USA; 3 University of Colorado Anschutz Medical Campus, Department of Bioengineering, Aurora, CO 80045 USA; 4 University of Colorado Anschutz Medical Campus, Department of Neurology, Aurora, CO 80045 USA; 5 Medtronic PLC, Neuromodulation Operating Unit, Minneapolis, MN 55432 USA; 6 University of Colorado Anschutz Medical Campus, Department of Psychiatry, Aurora, CO 80045 USA

**Keywords:** Diagnostic markers, Parkinson's disease

## Abstract

Oscillatory activity within the beta frequency range (13–30 Hz) serves as a Parkinson’s disease biomarker for tailoring deep brain stimulation (DBS) treatments. Currently, identifying clinically relevant beta signals, specifically frequencies of peak amplitudes within the beta spectral band, is a subjective process. To inform potential strategies for objective clinical decision making, we assessed algorithms for identifying beta peaks and devised a standardized approach for both research and clinical applications. Employing a novel monopolar referencing strategy, we utilized a brain sensing device to measure beta peak power across distinct contacts along each DBS electrode implanted in the subthalamic nucleus. We then evaluated the accuracy of ten beta peak detection algorithms against a benchmark established by expert consensus. The most accurate algorithms, all sharing similar underlying algebraic dynamic peak amplitude thresholding approaches, matched the expert consensus in performance and reliably predicted the clinical stimulation parameters during follow-up visits. These findings highlight the potential of algorithmic solutions to overcome the subjective bias in beta peak identification, presenting viable options for standardizing this process. Such advancements could lead to significant improvements in the efficiency and accuracy of patient-specific DBS therapy parameterization.

## Introduction

Subthalamic nucleus (STN) deep brain stimulation (DBS) for Parkinson’s disease (PD) has demonstrated favorable outcomes in ameliorating motor and nonmotor symptoms^1–3^. With advancements in imaging and connectomics, treatment efficacy and enhanced therapeutic benefit have become dependent on precise localization and selection of the optimal DBS contact configuration^4–6^. A myriad of studies has broadly identified the dorsolateral STN as the “electrophysiological sweet spot” for targeting symptoms of PD^6,7^. The proximity of stimulation to this region exhibits a positive relationship with improvement in motor performance^6–10^. The importance of optimized targeting and stimulation parameterization is demonstrated by the suboptimal effects of conventional non-adaptive, continuous high-frequency DBS.

Within the dorsolateral STN of patients with PD, local field potentials (LFPs) frequently exhibit increased oscillatory synchrony and elevated power in the beta frequency range of 13–30Hz^6,11–17^. Clinical evidence from human studies supports the growing consensus that LFP activity within this beta range is implicated in tonic movement^15,18,19^, is strongly correlated with rigidity and bradykinesia^20–22^, and that attenuation of beta power by dopaminergic medication and DBS results in symptom improvement^6,12–15,19,23–25^. Associations with symptom improvement are further supported by targeting the lower beta band (~13–20 Hz)^15,23,24,26–29^, although there are isolated reports of upper beta band (~20–30 Hz) activity governing freezing of gait^27,30,31^ and general lower limb activity^32^. As such, specific relationships between the upper/lower beta band and clinical outcomes are debated but nonetheless implicative for DBS therapies. Relatedly, adaptive DBS (aDBS) relies on biomarkers, predominantly the beta band, to adjust stimulation parameters based upon the degree of beta frequency modulation. Properties such as features within the beta frequency band among resting state LFPs and relative power changes are currently being implemented to guide aDBS^11,12,14,30^. As such, identifying optimal aDBS settings is essential to maximizing therapeutic efficacy.

**Fig. 1:**
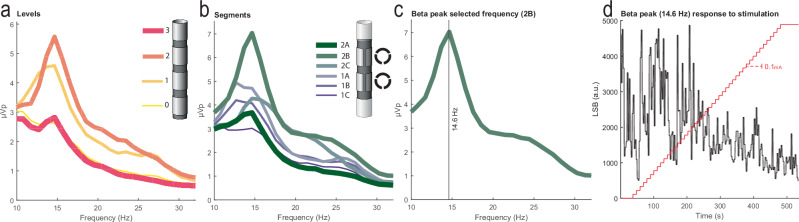
Visual representation of the current standard practice of using local field potential (LFP) power spectral density (PSD) information to identify an LFP peak within the beta frequency range (13–30 Hz) to track for PD DBS therapy. Example participant PSDs are displayed for all DBS (**a**) Levels and (**b**) Segments. **c** Peak selected frequency based on the highest beta peak power extrapolated from (**b**). **d** Beta frequency peak selected at 14.6 Hz in response to titration of DBS amplitude in 0.1 mA steps. Suppression onset appears to occur around 2.5 mA and remains stable up to 5 mA (*y*-axis scale is in least significant bits (LSB)).

Maximal amplitude beta peak (MBP) presence in LFP power spectral densities (PSD) were strongly related to the “electrophysiological sweet spot” for lead placement in 92% of patients undergoing DBS^6,33^. As such, several studies have corroborated the utility of PSDs to identify MBP to guide clinical decisions on DBS contact selection and parameterization in order to suppress beta power, which generally elicits clinical benefit^6,10,12,18,23,34^. While current clinical-commercial tools, including those from Medtronic^24^, Newronika^35^, and PINS^36^ permit spectral analysis, there remains a need to systematically quantify LFP peaks from PSDs [Fig. 1].

Clinical decision-making is thus augmented by efficiency in beta peak identification for accurate contact selection and parameterization. Currently, the standard practice uses bipolar sensing montages to determine optimal DBS contact placement^37^. This conventional practice details meticulous comparison of MBP between contact pairs, denoted by the largest voltage difference in pairs, which is largely reliant on trial-and-error and requires expertise in interpreting computed results^12,24^. To relieve this clinical burden and optimize the clinical workflow, an efficient peak detection algorithm could be integrated. Current efforts aim to standardize this process, and several algorithms have been proposed for detecting beta peaks, with varying degrees of complexity and generalizability. However, most of these studies perform peak detection based on subjective review requiring specialized knowledge and intervention^11,18,25,34,38^.

In this study, we evaluated objective algorithms for beta peak identification from implanted DBS electrodes. We observed acceptable concordance across observers, and despite subtle differences in algorithm success in classifying consensus peaks, many perform with low error. Importantly, algorithm performance varies by subject-specific PSD, and we introduce a novel monopolar sensing montage that improves LFP resolution of beta peak classification and clinical interpretability. Considering global and socioeconomic disparities in DBS access^39,40^ as well as heterogeneity in PD motor phenotypes reflected in diverse LFP power spectra^15,26,27,30,31^, our work could advance and broaden towards a streamlined, objective beta peak detection algorithm for identifying optimal contact configurations and stimulation parameters with greater feasibility alongside less reliance on exhaustive programming-guided intervention.

## Results

### Characterization of reviewer peak selection performance across all PSDs

**Fig. 2:**
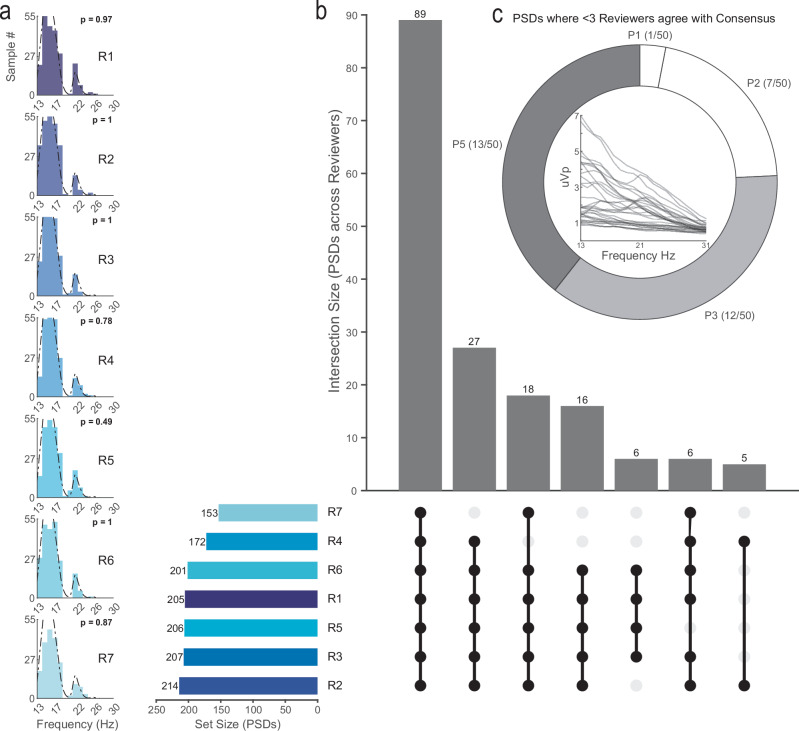
Characterization of reviewer peak selection performance across all PSDs. **a** Frequency distribution plots for all seven reviewers (R1–R7) with the peak consensus distribution superimposed (dotted line). A Kolmogorov–Smirnov two distribution test was used to compare each reviewer’s performance against the consensus distribution. **b** UpSet plot depicts the combination of reviewer groups that accurately identified the consensus peak for a set of PSDs. Horizontal bar chart depicts the total number of PSDs across sets in which the reviewer accurately identified the consensus peak. Vertical bar chart represents the frequency of set intersections (meaning the combination of reviewers agreeing upon a set of PSDs) ordered from the highest number of intersections (most common set combinations) to the lowest number of intersections (least common set combinations). The connected dot plots indicate the reviewers included in each intersection set (note that subsets of 4 or less are not shown; see Supplemental Fig. 1 for the full plot). **c** The donut frequency chart depicts the number of PSDs per participant that were correctly identified by less than half of the reviewers (3 or fewer). The inset line plot at the center of the donut chart illustrates the distribution of all PSDs (*n* = 33/250 PSDs from *n* = 4/5 participants) that met these criteria.

For this study, we evaluated the performance of seven independent expert reviewers in their subjective selection of the first and second (if applicable) beta peak frequency from fifteen PSD plots, each across five participants (both left and right hemispheres). As a group, the distribution of consensus peaks derived from all 250 PSDs was non-significantly different from the peak selection distribution of each individual reviewer, determined using a two-sample Kolmogorov–Smirnov test with the following *p*-values for each reviewer: R1: *p* = 0.97, R2: *p* = 1, R3: *p* = 1, R4: *p* = 0.78, R5: *p* = 0.49, R6: *p* = 1, R7: *p* = 0.87 (Fig. 2a); corroborated by similar p-values yielded from non-parametric rank-sum tests (see Supplemental Table 2). At a minimum, there are two factors that could affect the derivation of the consensus peak quality: (1) a subset of reviewers performed substantially worse on accurate classification of the consensus peak, or (2) a subset of PSDs was more challenging to classify across reviewers. To assess these two factors, we used an UpSet plot (Fig. 2b)^41^ which is a method to visualize and quantify the number of intersections between sets. Both of these issues are not mutually exclusive and could contribute to the consensus peak accuracy. Summarizing across reviewers, regarding the number of PSD sets in which an individual reviewer correctly identified the consensus beta peak, highlights two reviewers that made accurate selections for fewer than 70% of the PSDs (R7 61% and R4 69%) while the remaining reviewers made accurate selections on greater than 80% of the PSDs. In the main summary across reviewer and PSD set intersections, all reviewers correctly identified the consensus beta peak on 35% of the PSDs. To determine if there was a subset of PSDs that was difficult to accurately assess across reviewers, we identified the participant ID of PSDs for which fewer than four reviewers accurately identified the consensus beta peak (Fig. 2c). We found that two participants contributed approximately 25% of their PSDs to this low-consensus group (Participant 3 and Participant 5). Fewer problematic PSDs were observed for Participant 1 (2%), Participant 2 (14%), and Participant 4 (0%).

### Characterization of algorithm peak selection performance across all PSDs

**Fig. 3:**
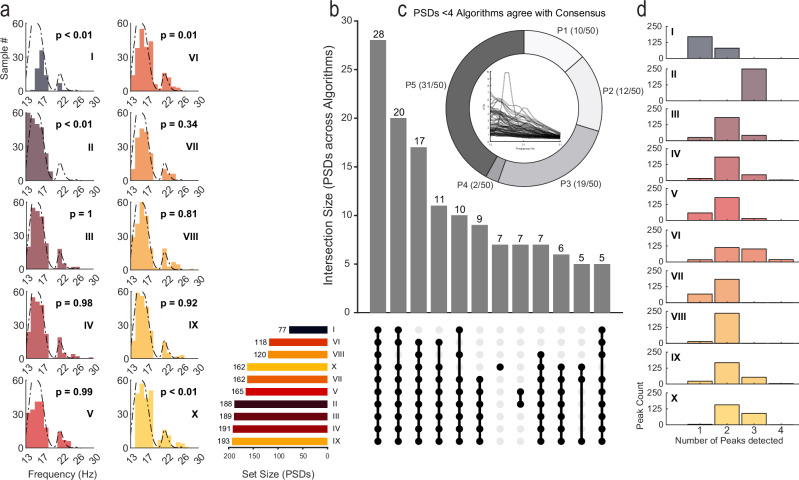
Characterization of Algorithm peak selection performance across all PSDs. **a** Frequency distribution plots for all ten algorithms (I–X) with the peak consensus distribution superimposed (dotted line). A Kolmogorov–Smirnov two-distribution test was used to compare each algorithm’s performance with the consensus distribution. **b** UpSet plot depicts the combination of algorithm groups that accurately identified the consensus peak for a set of PSDs. Horizontal bar chart depicts the total number of PSDs across sets in which the algorithm accurately identified the consensus peak. Vertical bar chart depicts the number of intersections between sets (meaning the combination of algorithms agreeing upon a set of PSDs) ordered from the highest number of intersections (most common set combinations) to the least common. The lower connected dot plots indicate the algorithms included in each intersection set (note that subsets of 4 or less are not shown; see Supplemental Fig. 2 for the full plot). **c** The donut frequency chart depicts the number of PSDs per participant that were correctly identified by less than half of the algorithms (4 or fewer). The plot at the center of the donut chart depicts all PSDs (*n* = 74/250 PSDs from *n* = 5/5 participants) that met these criteria. **d** Bar charts, one for each algorithm, summarize the number of one, two, three, or four peaks detected across all PSDs.

Following the derivation of expert-defined consensus beta peaks for all 250 PSDs, we sought to evaluate the performance accuracy for ten algorithms used in the literature for detecting PSD peaks. We did not impose any a priori restrictions on the maximum number of peaks that algorithms could detect. Furthermore, the algorithms were not configured to guarantee the detection of at least one peak, meaning it was possible for an algorithm to fail to detect any peaks. Comparing the consensus-derived peak distribution (for the first peak detected) with that of each individual algorithm revealed that four algorithms (I, II, VI, X) produced peaks with distributions significantly different from the consensus (two-sample Kolmogorov–Smirnov test, *p* < 0.01). In contrast, six algorithms (III, IV, V, VII, VIII, IX) yielded peak distributions similar to the consensus, as shown in Fig. 3a, corroborated by similar p-values yielded from non-parametric rank-sum tests (see Supplementary Table 3). To evaluate algorithm accuracy in detecting the consensus peak, we adopted an approach similar to our assessment of reviewer performance. We visualized the performance of individual algorithms across PSDs and analyzed the overlap among algorithms that successfully identified the peak. This analysis is shown in Fig. 3b. We observed that algorithms exhibited substantial variability in accuracy, shown in the lower horizontal bar chart in Fig. 3b, which summarizes individual algorithm accuracy across all PSDs. Three algorithms performed below 50% accuracy (I = 30%, VI = 47%, VIII = 48%), three performed below 75% accuracy (X = 64%, VII = 64%, V = 66%) and four algorithms performed above 75% accuracy (II = 75%, III = 75%, IV = 76%, and IX = 75%). The vertical bar chart of the UpSet visualization in Fig. 3b depicts the main summary across algorithm and PSD set intersections. The greater number of possible intersection sets resulted in a greater diversity of set relationships in the plot. In general, all ten algorithms correctly identified the consensus beta peak on 11% of the PSDs. Following the approach we used in the reviewer performance assessment, we evaluated whether a subset of PSDs was difficult to accurately assess across algorithms. For this analysis, we quantified the participant ID of PSDs for which fewer than five algorithms accurately identified the consensus beta peak (Fig. 3c). We found that all participants contributed a subset of PSDs that were challenging for half or more of the algorithms. The following participant PSD contributions were observed: participant 5 was the most challenging (62% PSDs, where < 4 algorithms match the consensus peak), followed by participant 3 (38%); participants 1 and 2 exhibited moderate difficulty with 20% and 24% respectively, and participant 4 again exhibited the least difficulty across algorithms (4%). All PSDs with successfully detected peaks by less than four algorithms were represented within the center of the donut chart (Fig. 3c). Algorithms also exhibited substantial variability in the number of peaks detected across PSDs (Fig. 3d); most algorithms predominantly detected two peaks per PSD.

### Overall algorithm performance for detecting reviewer consensus beta power peaks

**Fig. 4:**
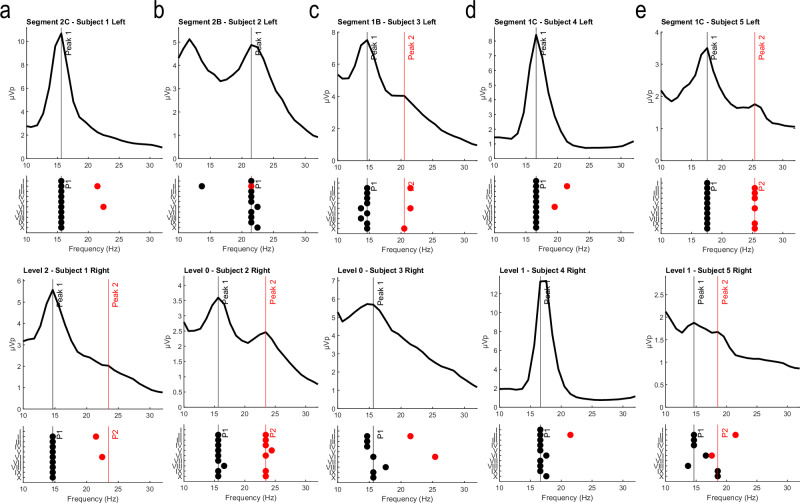
Case examples of algorithm performance in detecting reviewer consensus beta power peaks for each participant. **a**–**e** represents Participants 1–5. The top row depicts a representative PSD from the DBS electrode segment determined to have the highest peak beta power on the left hemisphere and the bottom row depicts a representative PSD from the DBS electrode level determined to have the highest peak beta power on the right hemisphere. The vertical line(s) in each PSD plot indicates the consensus peak for Peak 1 (black) and Peak 2 (red). If no reviewer identified a 2nd peak, then no red line was depicted for that PSD. Instances in which the dot is not aligned with the consensus line indicate a discordant evaluation by an algorithm. Algorithm performance is represented in the plot below each PSD. Each dot represents the assessment for each algorithm along the *y*-axis. If an algorithm identified a second peak when no Peak 2 consensus was determined, then red points will not align with a vertical red peak line.

**Fig. 5:**
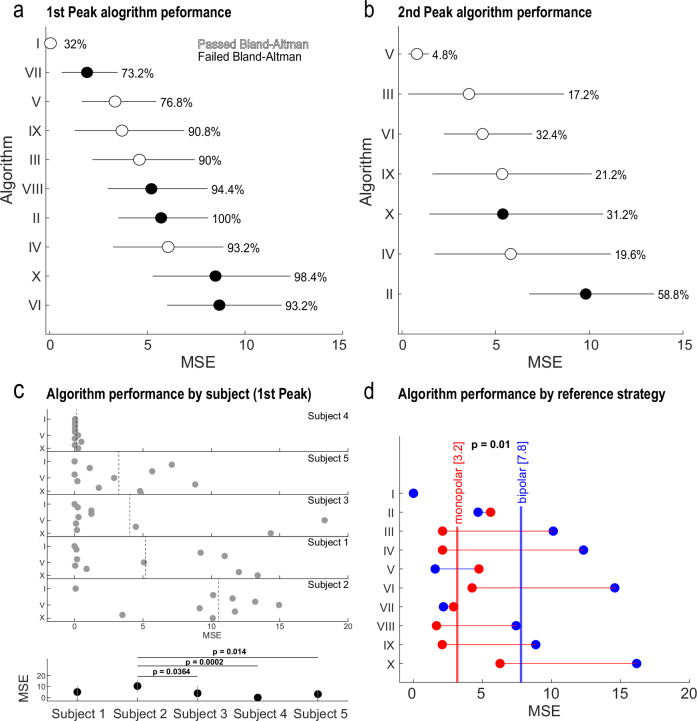
Overall algorithm performance in detecting reviewer consensus beta power peaks. **a** Accuracy assessment of algorithm performance based on mean squared error (MSE) for the first peak identified across PSDs. Algorithms were ranked by performance and are ordered along *y*-axis from highest to lowest accuracy. Algorithms that obtained poor agreement based on Bland-Altman analysis were noted by filled circles, and algorithms that obtained high agreement were denoted by empty circles. Circles represent mean MSE, and horizontal lines indicate the MSE standard deviation. The percentage value adjacent to each circle indicates the proportion of PSDs that were evaluated by the algorithm. **b** Accuracy assessment of algorithm performance based on MSE for the second peak identified across PSDs with equivalent formatting to that in (**a**). **c** Top plot depicts the average MSE for each algorithm separated by participant. Participants were ranked from highest to lowest accuracy. Dotted lines in each participant-specific plot denote the mean MSE across algorithms. The lower plot summarizes the statistical comparison of MSE across participants using an ANOVA (*F*(4,45) = 6.04, *p* = 0.0006). **d** Accuracy assessment of algorithm performance based on the reference strategy. PSDs were separated by monopolar (red) and bipolar (blue) referenced recordings. The connecting line between each algorithm comparison indicates which referencing strategy exhibited the highest accuracy (lowest MSE). Statistical comparison between monopolar and bipolar referencing demonstrated that monopolar was more accurate than bipolar assessed PSDs (*p* = 0.01).

The main goal of the study was to assess algorithm performance in detecting expert-defined beta peaks in PSD representations of LFP data. We evaluated the performance of individual algorithms using two approaches. First, we used a Bland-Altman test to assess the agreement between the expert consensus peaks and the first peak identified by each algorithm. A *t* test on the mean difference between the two measurement methods was used to determine if there was a significant difference from zero, indicating a systematic bias between the measures. In this case, a *p*-value less than alpha (0.05) would suggest a possible systematic bias, indicating that those algorithms may be less reliable. Second, we quantified algorithm accuracy by evaluating the mean squared error (MSE) between the consensus peaks and the first peak derivations for each algorithm. Representative results showing the comparison of consensus and algorithm-detected peaks are shown for all five participants in Fig. 4. (The top row depicts PSDs from segments on the left hemisphere that exhibited the highest peak beta, and the bottom row depicts PSDs from levels on the right hemisphere that exhibited the highest peak beta; all PSDs were derived from monopolar referenced recordings). Of greatest interest was the first peak detected, as all reviewers selected up to two peaks, with the first predicated as the main peak. For the first peak (Fig. 5a), we found that five of the ten algorithms passed the Bland-Altman test (I, V, IX, III, and IV) while five failed to pass (VII, VIII, II, X, VI). In general, there was a correlation between passing the Bland-Altman and having a lower MSE, with four algorithms that passed the Bland-Altman test exhibiting the lowest MSE. However, we found that MSE could be impacted by the number of PSDs in which any peak was detected. For example, algorithm I, which failed to identify any peak for 67% of the PSDs, had the lowest MSE; this algorithm was the most conservative algorithm in peak detection. All other algorithms successfully identified at least one peak in over 73% of the PSDs. We observed a similar phenomenon when assessing accuracy for the second peak (Fig. 5b). Only seven of the ten algorithms detected a second peak, and these detections occurred at a lower frequency across PSDs (average of 26% of PSDs per algorithm). The increased stringency in second-peak detection resulted in lower MSEs, similar to the accuracy of algorithm I for first-peak classification.

The cohort of PD participants (*n* = 5; 10 hemispheres) used for this study was representative of the heterogeneity in PSD morphology (e.g., peak number, slope, peak magnitude). Despite the inherent variability in overall PSD structure, both reviewers and algorithms were generally able to agree on the identified beta peak(s) in many cases. We directly evaluated the participant-based variability in algorithm performance to determine whether specific participants exhibited more challenging PSD morphologies for beta peak detection. In Fig. 5c, we arranged participants from highest to lowest accuracy based on the average MSE across algorithms, revealing notable differences that reinforce earlier results; for example, participant 4 exhibits less variability in MSE than participant 2. Applying an ANOVA to assess participant MSE variability demonstrated a significant difference in accuracy between participants (*F*(4,45) = 6.04, *p* = 0.0006). Post-hoc tests revealed that participant 2 was significantly different from participants 3 (*p* = 0.04), 4 (*p* = 0.0002), and 5 (*p* = 0.01). This inter-subject heterogeneity was additionally reflected in dynamic threshold values used for algebraic algorithms such as method III. Therein, divisor values were iteratively parsed to determine optimal values, which varied between subjects and within subjects (i.e., between STN hemisphere). The following were the highest accuracy divisor value(s) per hemisphere: [1L: 54.57-100; 1R: 59.20-100; 2L: 25.62–100; 2R: 13.42-100; 3L: 19.34-100; 3R: 20.82–100; 4L: 0.55-100; 4R: 0.30–100; 5L: 41.71-100; 5R: 56.16-100] which converged on the most optimal value of 14.46 for the present sample.

In this study, PSD plots were generated using two referencing strategies: bipolar, which references between neighboring contacts on the same lead, and monopolar, which takes measurements between the electrode being sensed on the DBS lead to a common reference electrode on the other implanted DBS lead. We define the monopolar approach based on the concept that this strategy allows for the isolation of activity from a single contact or set of contacts (i.e., combined segments) on an implanted DBS lead. We sought to determine whether the monopolar approach resulted in greater isolation of peak activity and, thereby, greater accuracy in peak identification. In Fig. 5d, we directly compared the PSD data derived from monopolar and bipolar contact configurations using a paired *t* test. We observed that PSDs generated by monopolar-referenced contact configurations resulted in greater accuracy compared to bipolar-referenced PSDs (monopolar mean MSE = 3.2; bipolar mean MSE = 7.8; *p* = 0.01).

### Accuracy of top performing algorithms to predict contact level of stimulation at 3 months follow-up

Finally, we evaluated whether the top performing algorithms, ranked by accuracy in detecting expert reviewer-derived consensus beta peak(s), would have clinical utility. We replicated this approach by assessing how well the top three performing algorithms predicted the contact level of stimulation clinically determined and used at 3-months follow-up. We used three criteria to determine the top performing algorithms: (1) pass the Bland–Altman test while retaining the null hypothesis, (2) detect at least one peak from 75% of the PSDs, and 3) accuracy rating of MSE < 5, which was the median MSE across all ten algorithms. Per our evaluative criteria, three methods (III, V, and IX) were deemed most satisfactory. Of note, methods III and V were both algebraic methods such that a dynamic threshold was used to identify relevant peaks using properties of individual PSDs. Method IX employed the same algebraic thresholding approach as method III, albeit applied to a PSD whose (1) $\frac{1}{f}$ component was subtracted; thus, it was classified as elemental decomposition yet drew heavily from algebraic algorithms. As such, all three satisfactory methods overlapped in the use of individualized, dynamic peak amplitude thresholding procedure, pointing to a promising, standardizable peak detection strategy. At 3-month follow-up, seven of the ten hemispheres were programmed either at a single level or, if programmed across levels, had greater than 50% stimulation fractionated to one level. These seven hemispheres were used for the comparison. When comparing the level selected by the three top performing algorithms based on the max beta peak identification, we observed the following accuracies in predicting the contact level for each hemisphere at 3-months follow-up: V = 71%, IX = 86%, and III = 100%.

## Discussion

In this study, we evaluated the utility of a set of algorithms in matching the performance of experts in identifying therapeutic frequency peaks in PSD derived from LFP recordings in patients with PD. A primary goal was to assess objective clinical decision-making strategies for improved efficiency and efficacy in DBS programming. DBS therapy management relies on accurate evaluation of physiological responses to stimulation, including electrophysiological signals at the site of stimulation. In PD, real-time measures of LFP show that modulation of STN beta power correlates with various clinical responses^18,23,42,43^.

Clinicians have employed a variety of biomarkers to guide lead placement and stimulation titration for DBS. Neuroimaging-guided approaches for DBS programming have yielded non-inferior adverse motor symptom attenuation compared to conventional, monopolar review techniques^44,45^. Other strategies have employed methods drawing from electric fields in tandem with functional MRI signatures along cortico-basal ganglia-thalamo-cortical circuitry to inform targeting and stimulation optimization^4^. More recently, evoked potentials have also demonstrated utility as a viable biomarker, particularly with emerging advancements in stimulation parameterization^46^. However, advances in therapeutic delivery have identified LFP features as robust, widely supported biomarkers for DBS programming and aDBS control system feedback^16,24,47–51^. While extensive evidence demonstrates that LFP features within the beta frequency range are clinically relevant and reliable biomarkers of PD state, not all patients exhibit exaggerated beta activity or a pronounced beta peak, given the significant heterogeneity in electrophysiological dynamics and clinical profiles across PD patient populations. Beyond the scope of this manuscript, LFP biomarkers within the low frequency (4–12 Hz), gamma frequency (35–200 Hz), and high-frequency oscillation (HFO, >200 Hz) ranges are also projected candidates for LFP-guided DBS programming and aDBS control system development. Reviews of electrophysiological signatures in these canonical frequency bands can be found in the following selected publications^29,51^.

The current approach for evaluating beta power to guide DBS therapy in PD relies on spectral analysis. While current clinical-commercial tools facilitate spectral analysis and can be used to identify and monitor clinically relevant signals of interest, identification of validated frequency-based biomarkers (e.g., MBP, which may provide the most efficacious therapeutic outcome) relies on the subjective experience of a clinician^31^, which lacks objectivity, relies on specialized knowledge, and may be subject to bias. Our focus on the primary peak within the low beta frequency range for guiding STN DBS parameter tuning for PD is predicated on the high reliability of this biomarker for predicting therapeutic response^19,49,51,52^. We assessed 250 PSDs from 5 participants and 10 hemispheres and observed high reviewer agreement (Fig. 2), with all reviewers exhibiting peak selections that did not significantly differ from the consensus peak distribution. Although reviewers exhibited high agreement for a significant proportion of PSDs, a subset of PSDs was challenging to assess across reviewers (Fig. 2c). In contrast, algorithm performance varied significantly, either in overall performance across PSDs or with respect to specific subsets of PSDs that were challenging to match with the consensus. For example, participant 2 resulted in many mismatches across algorithms (Fig. 5c). To assess for systematic bias, we used the Bland-Altman analysis using a threshold of *p* > 0.05 as the first method to identify accurate algorithms that had acceptable peak identification accuracy when compared to expert-defined (Table 1, Fig. 5a, b). We observed that more conservative algorithms (i.e., algorithms that failed to detect any peak in specific PSDs) exhibited high agreement with the consensus when peaks were identified. An algorithm that performed well on only a subset of PSDs may not be generalizable; therefore, we eliminated algorithms that failed to render a peak selection for greater than 30% of PSDs used in the study. In addition, we set the accuracy threshold to the median performance across the ten algorithms (MSE = 5). Notably, the three algorithms (III, V, and IX) that met performance criteria successfully predicted the therapeutic level of stimulation clinically determined for 7/10 hemispheres at 3-month follow-up. These methods exhibited shared qualities; methods III and V were algebraic methods such that a dynamic prominence threshold using PSD properties was employed to identify relevant peaks. Of note, method IX employed the same algebraic thresholding approach as method III, albeit applied to a PSD whose (1) $\frac{1}{f}$ component was subtracted; thus, it was classified as elemental decomposition yet drew heavily from algebraic methodologies (Table 1). As such, these three algorithms overlapped in the use of dynamic peak amplitude thresholding, pointing to a promising mathematical mechanism undergirding a potentially standardizable, universal peak detection strategy.

**Table 1:** Descriptions of each method and thresholding approaches

Method		Description	Alternative threshold	Bland–Altman *p*-value (MSE) [95% Conf. Int]	
Absolute	**I** [Jimenez-Shahed^60^.; Vaou et al. ^50^]	*Medtronic’s Percept™ PC BrainSense Survey (BSS) uses a static peak detection threshold of* > *1.1µVp. Other peaks are subjectively determined*.		**I** 0.566 (0.035) [0.000, 0.083]	
	**II** [Giannini et al. ^26^]	*Local maxima are identified within the lower (13-20* *Hz) and upper (20-30* *Hz) beta ranges*.		**II** 8.820^-10^ [3.608, 8.008] (38.0630)	
Algebraic	**III** [Darcy et al. ^33^]	*With normalized PSD signatures, a dynamic threshold is set for findpeaks’ MinPeakProminence of median(PSD*_*norm*_*) / divisor. Iteratively run until an optimal divisor value is found*.	**IV** [Parameshwaran and Thiagarajan^61^.; Weber et al.^62^] *Dynamic threshold of std(PSD*_*norm*_) * multiple is used.	**III** 0.379 (4.590) [2.184, 7.478]	**IV** 0.091 (6.061) [3.258, 9.278]
	**V** [Plate et al., 2021; de Solages et al. ^63^]	*Beta peak is defined as a local elevation of power such that a peak’s 4-6 adjacent points must be 20% greater than the means of the outer 8-12 points*.		**V** 0.617 (3.342) [1.656, 5.374]	
Elemental Decomposition	**VI** [Donoghue et al., 2020; Wiest et al. ^54^]	*Power spectra are deconstructed into aperiodic (1/f trend) and periodic components using a series of Gaussian fits. Peaks and prominences are fit based on these fitted models*. [2, 12] (*peak_width_limits*), infinity (*max_n_peaks*), 0.1 (*min_peak_height)*, 2 (*peak_threshold)*, ‘fixed’ *(aperiodic_mode*).	[Wilson et al. 2022] *The aperiodic component is treated as temporally dynamic instead of static, and averaged before peak identification*.	**VI** 4.140^-6^ (8.685) [6.143, 11.667]	
	**VII** [Weber et al. ^62^]	*The aperiodic component is defined by the linearized Lorentzian function* ${10}^{b}* \frac{1}{(k+\,{F}^{x})}$ *where b = offset; k = knee parameter (k* = *0); F = frequency vector; x = aperiodic exponent. This is subtracted from the PSD signature. A threshold of mean(PSD)* + 3*SD(PSD) is used to identify peaks/	**VIII** [Parameshwaran and Thiagarajan, 2019] *An alternate dynamic threshold of mean* + *1* *SD is used to generate a Heaviside-like function with which peak indices are defined*.	**VII** 0.002 (1.907) [0.541, 3.739]	**VIII** 0.010 (5.200) [2.822, 7.982]
			**IX** [Darcy et al.^33^] *An alternate dynamic threshold approach from III is used on non-normalized-PSD*.		**IX** 0.178 (3.697) [1.434, 6.394]
	**X** [Wang et al. ^17^]	*log(PSD) is fit to a 5th* *order polynomial using the local minima in the lower frequency range (3–5* *Hz) and values above the beta range as a 1/f proxy. The difference between 1/f proxy and the log(PSD) is fit to a Gaussian and peaks identified*.		**X** 3.470^−8^ (8.486) [5.132, 12.291]	

Our exploration reveals that monopolar referencing may significantly improve the resolution of beta peak identification (Fig. 5d, [*t*(9) = −2.7687, *p* = 0.0109]). Findings spotlight practical candidates for objective beta peak detection using a high-resolution monopolar configuration with potential utility in empirically and efficiently determining optimal starting points for DBS therapy. Further refinement of a method utilizing this monopolar referencing strategy via a larger sample and diverse representation of PSD signatures could offer a quantitative, generalizable peak detection approach that is independent of subjective review. Variable peak determination presents important implications for clinical decision-making. Of note, certain methods suggest peak bimodality in a subset of patients, while others do not, as illustrated by distribution differences (Fig. 3d). For example, method VI employed elemental decomposition to subtract out the (1) $\frac{1}{f}$ aperiodic component characteristic of biological systems, which could have underestimated lower frequencies’ power compared to higher frequencies, resulting in performance bias^53^. Incidentally, emerging work in animal^54^ and human^55^ models has identified this aperiodic component as a promising biomarker of generalized motor symptom severity, potentially pointing to another useful biomarker. In general, our approach predominantly identified a more prominent peak within the low beta range. Given that low beta (~13–20 Hz) power, in comparison to high beta (~21–30 Hz) power, is more significantly correlated with bradykinesia severity^14^ and more systematically suppressed in response to STN DBS therapy^23^, peak detection algorithms tuned for low beta peak identification could augment clinical programming efficacy. However, the algorithms we tested could have been tuned for peak detection in the high beta range, which has been implicated in PD phenotyping; high beta activity is more pronounced in akinetic-rigid compared to tremor-dominant motor subtypes^31,56^. Recent tractography work suggests that high beta may also reflect a regionally segregated informational pathway for lower limb cortical-subcortical coherence, observed in freezing of gait^11,27,29,31,32^, and others ascribe high beta activity to cognitive features of PD^13^ or typical neurophysiology^26^. If high beta is associated with PD pathophysiology, uncovering bimodality may be crucial for symptom management. Thus, it is imperative that clinicians converge to a consensus on how to identify clinically relevant beta-band peaks in LFP power spectra.

Limitations of this study include small sample sizes, heterogeneity of different LFP montages (which is also a strength), reliance on reviewer-determined ground truth, and a narrow focus on the beta frequency band. Heterogeneity across LFP montages resulted in variable processing times for select methods. For example, method III, one of the best-performing methods, used 10,000 iterations per subject’s hemisphere PSD signatures to identify optimal divisor values. Using a commercially available personal laptop (i.e., subpar RAM and computing efficiency), parsing 10,000 iterations and plotting accuracy charts took 282.35 s ± 48.42 s (4.70 min ± 0.80 min), with a range of [235.50 s (3.92 min), 369.68 s (6.16 min)]. In addition, several studies have noted the clinical utility of LFP features within the theta^30,57,58^, alpha^29,51^, and gamma^12,29,32^ frequency ranges, and the algorithms tested in this study may or may not prove useful for these spectral bands. Nevertheless, this study compared theoretical results to applied clinical outcomes to evaluate our methods’ practical clinical utility. Notably, in our assessment of objective, algorithmic peak detection approaches, results revealed that top-performing algorithms predicted the optimal contact level clinically determined and programmed by expert neurologists in >70% of participant hemispheres. Selection of specific algorithms based on study design and available resources in future work may improve data collection consistency and interpretation across studies. Another limitation to current peak algorithms is processing time. While not the aim of the study, an algorithm that detects peaks within seconds would theoretically be more time efficient than subjective examination of the entire frequency spectrum and selecting peaks.

By elucidating the ambiguity in current approaches and highlighting promising objective beta peak identification methods, this study points toward practical avenues for the advancement of standardized, efficient, and data-driven clinical decision-making strategies. The resulting insight may contribute to better-targeted stimulation delivery and optimized clinical benefit. Future studies may choose to derive approaches from methods III, IX, or V and apply to an expanding, diverse database of PD LFPs or related movement disorders to augment generalizability, given their translational utility in DBS programming, as demonstrated by acceptable peak detection and contact level prediction accuracy in our sample. Therein, algebraic methods (e.g., III, IX) could be modified by adjusting the looping function used to determine optimal divisor values. For example, one may choose to decrease the resolution of iterations (i.e., 10,000 down to 1000 using [0:0.1:100 instead of 0:0.01:100] which improves processing time by 90.9% on a personal laptop. Similarly, a growing database of subjects’ PSD signatures could be amassed to determine optimal divisor values with increasingly generalizable samples; when a divisor value is selected after iterative exploration, detecting peaks and determining algorithm accuracy on a personal laptop is achieved in 0.5 s. Insights produced from this study may also inform LFP biomarker-driven control strategies for advanced, patient-specific aDBS therapy. In addition, pioneering work towards a streamlined, standardized objective peak detection algorithm could be of benefit for under-resourced clinics across the world or telemedicine interventions by providing access to efficient, computerized peak detection algorithms without oversight from specialized movement disorders neurologists or other members of the care team.

## Methods

### Data collection

We collected LFP data from SenSight^TM^ directional DBS leads (Model B33005, Medtronic; Minneapolis, MN USA) implanted in the STN in five participants with PD (*N* = 10 hemispheres). All participants were recruited from the University of Colorado Anschutz Medical Campus Advanced Therapies in Movement Disorders Program. For all subjects, the study was conducted at the initial DBS programming session (27.4 ± 6.1 SD; days post-lead implant), medications were held for 12 hours overnight prior to the study visit. All data collection and study conditions were conducted in the OFF medication and OFF stimulation state (see Supplemental Table 1). LFP data can be collected using Percept^TM^ PC BrainSense^TM^ features using a bipolar sensing configuration. In addition, the investigational-use Percept^TM^ SP Advanced Sense research system was used to collect LFP data to enable a monopolar sensing configuration through a temporary software unlock. The implanted neurostimulator maintained standard commercial operation, and therapy was not affected while using the research system. This temporary unlocking of additional sensing configurations was used only in the clinic under movement disorder neurologist supervision. LFP data were collected from one hemisphere per session. A sensing montage that iterated through predetermined configurations was performed to wirelessly stream LFP data at 250 Hz for 55 s per recording. All recordings passed through two low-pass filters set to 100 Hz and two high-pass filters set to 1 Hz (both bipolar and monopolar contact configurations). The following configurations were collected: Bipolar: all possible segment pair configurations (*n* = 9), all possible level pair configurations (*n* = 6); Monopolar: all individual segments (*n* = 6), all individual levels (*n* = 4). As such, this included the following monopolar: 0, 1 ABC, 1A, 1B, 1 C, 2 ABC, 2A, 2B, 2 C and 3, and bipolar configurations: 1A-1B, 1C-1B, 1C-1A, 2B-2A, 2C-2B, 2C-2A, 2A-1A, 2B-1B, 2C-1C, 3-2ABC, 3-1ABC, 1ABC-2ABC, 1ABC-0, 2ABC-0, 3-0. Three trials of each montage were collected to account for variability. Our study was carried out in accordance with the Colorado Multiple Institution Review Board (COMIRB # 21-4938) and the Declaration of Helsinki, with written informed consent obtained from all study participants.

### LFP postprocessing

Postprocessing and statistical analyses were performed using MATLAB R2023a. Periodograms were generated using *pwelch* with a Hanning window, 50% overlap, and 256 discrete Fourier transforms (0.9766 Hz resolution). Differences between session-level PSDs within each hemisphere were not statistically significant from one another per one-way ANOVA and Tukey’s HSD post-hoc tests (*p*_STN_ = 0.9294 ± 0.1372). As such, PSDs were averaged across the three sessions per contact, yielding a total of 250 PSDs obtained across five subjects (*n* = 50 per subject, *n* = 25 per hemisphere therein).

**Fig. 6:**
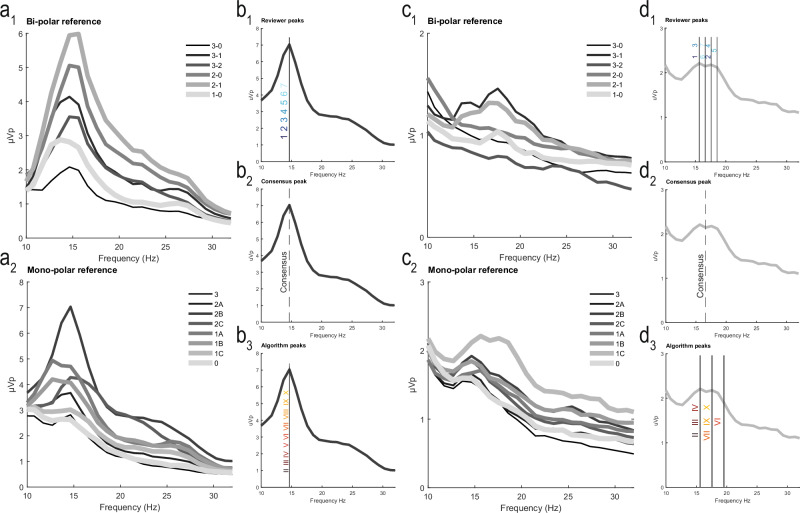
Example of power spectral density (PSD) plots, reviewer-selected peaks, the reviewer consensus peak, and the algorithm-selected peak(s) in two representative participants. A) PSDs of local field potential (LFP) recordings from all contacts, using both A_1_) bi-polar and A_2_) mono-polar referencing in a representative participant with an apparent beta peak (note that a subset of the PSDs is displayed in the A_1_ and A_2_); B) Peak determination by B_1_) Reviewers, B_2_) Reviewer consensus, and B_3_) Algorithms for the PSDs displayed in panel A. In this represented participant, there was clear agreement between reviewer consensus and algorithm performance C) PSDs of LFP recordings from all contacts, using both C_1_) bi-polar and C_2_) mono-polar referencing in a representative participant with a less obvious beta peak; D) Peak determination by D_1_) Reviewers, D_2_) Reviewer consensus, and D3) Algorithms for the PSDs displayed in panel C. PSDs for this participant resulted in less agreement between consensus and algorithm performance highlighting the ambiguity currently encountered in clinical and research practice.

Expert visual inspection of the PSDs and peak identification, as is performed in standard practice, was used to compare algorithms. A graphical user interface (GUI) permitted each reviewer to select up to two peaks. The GUI collected the x-axis frequency values (Hz) that were selected by the reviewer. The following strategy was used to derive the consensus peak frequency for each PSD: First, the proportion of all selected frequencies was computed. If one selected peak frequency exceeded 50%, it was set as the consensus frequency. If no selected frequency exceeded 50%, the median frequency was computed and selected as the consensus frequency. This approach for consensus derivation yielded 98.4% congruence. Figure 6 depicts the process that was used to compare reviewer-derived consensus peaks for both bipolar and monopolar examples to highlight the challenges of beta peak identification.

Ten existing or exploratory beta peak detection algorithms were selected from recent literature (published within the past ~10 years at the time of publication). Algorithms were heuristically categorized as absolute, algebraic, or elemental decomposition.

*Absolute algorithms*. Absolute implied that the value(s) within the algorithm did not change based on PSD properties. The Medtronic Percept^TM^ PC detection algorithm (I), as described in ref. ^59^ and used in refs. ^50,60^, utilized a >1.1µVp threshold to identify a singular MBP in non-normalized PSDs given the absolute threshold. Other clinically relevant peaks were traditionally determined subjectively. As this algorithm was modified to be objective where a second peak is seldom identified given the stringent threshold, it was subject to a high false-negative rate. The second method (II), as employed in ref. ^26^, identified local maxima within the lower beta band (13-20 Hz) and upper beta band (21-35 Hz), thus assuming bimodality. The only alteration made from its original methodology was the absence of PSD normalization, which was chosen as signal morphology is not altered and the algorithm solely searches for maxima within two frequency bands, so normalization would prove an extra, unnecessary step.

*Algebraic algorithms*. Algebraic implied that the algorithm fluctuated based on numeric properties of the PSD. For example, the third algorithm (III) was adapted from an approach described in ref. ^33^. Herein, using normalized PSDs, a dynamic threshold was set for the MinPeakProminence parameter within MATLAB’s *findpeaks* function, defined as equation (2) $\frac{{median}({{PSD}}_{{norm}})}{{value}}$, where the a priori defined value conventionally ranged from 4 to 12. However, as these divisor values were inadequate in our sample, our adapted algorithm employed an optimized value found by iteratively running 10,000 potential values ranging from 0 to 10 until the greatest overlap with the consensus ground truth was achieved. This choice underscores the diversity of PSDs reflecting equally heterogeneous PD symptom profiles^15,26,27,30,31^. The fourth algorithm (IV) was similarly adapted from^33^ such that the aforementioned iterative optimization procedure was used on normalized PSD, but instead on a dynamic ‘MinPeakProminence’ parameter threshold drawing from^61,62^ defined as equation (3) ${stdev}\left({{PSD}}_{{norm}}\right)\times {value}$. Lastly, the fifth method (V) was described in ref. ^63^ and employed in ref. ^27^. In this unaltered method, a beta peak was defined as a local elevation in power in non-normalized PSD, in which the 4-6 points defining a peak were at least 20% greater than the means of the 4-6 points beyond either tail (therefore 8-12 points) of this local elevation.

*Elemental decomposition algorithms*. This subtype considered the aperiodic and periodic components of PSDs. For example, in the sixth method (VI), as described in ref. ^53^ and used in ref. ^54^, non-normalized PSDs were deconstructed into aperiodic (1) ($\frac{1}{f}$ trend) via a linearized Lorentzian function and periodic components using a series of Gaussian fits. Peaks and prominences were then further fit based on these Gaussian and Lorentzian models via several ancillary mathematical transformations. From these series of model fits, parameters were defined per PSD to detect peaks. The seventh method (VII)^62^ similarly used non-normalized PSD signatures, defining the aperiodic component as a linearized Lorentzian function (4) ${10}^{b}\times \frac{1}{(k+\,{F}^{x})}$, where b = offset, k = knee parameter (in this case, k = 0), F = frequency vector, and x = aperiodic exponent. This aperiodic component was subsequently subtracted from the modified PSD, and a threshold modeled by equation (5) ${mean}({{PSD}}_{{modified}})+(3\times {stdev}({{PSD}}_{{modified}}))$ was used to identify peaks via MATLAB’s *findpeaks* function. In the eighth method (VIII), elemental decomposition was performed similarly to method VII such that non-normalized PSD were used, though an alternative dynamic threshold was employed from^61^. Instead of using *findpeaks*, this Heaviside-like threshold was defined by equation (6) ${mean}({{PSD}}_{{modified}})+{stdev}({{PSD}}_{{modified}})$, from which peak indices were defined. Following the theme of changing thresholding equations, the ninth method (IX) also employed the same elemental decomposition as method VII, but instead defined the *findpeaks* threshold for the modified PSD as that of method III^33^; therefore, in congruence with method III, normalized PSD were used for this method. Lastly, the tenth method (X) defined each periodic element in a different fashion. Herein, the logarithmic transform of the PSD as opposed to a normalized PSD signature was fit to a 5th order polynomial using the local minima of the lower frequency range (3–5 Hz) and values beyond the beta range as bounds for a (1) $\frac{1}{f}$ aperiodic component proxy. The difference between this (1) $\frac{1}{f}$ aperiodic component proxy and the (7) ${\log }_{{PSD}}$ was fit to a Gaussian model, and peaks were identified thereafter using the default *findpeaks* function^17^.

### Statistical analyses

We evaluated the performance of individual reviewers and algorithms against the consensus peak distribution for the first peak using a 2-sample Kolmogorov-Smirnov test. ANOVA with post-hoc tests were used to highlight between-subjects performance differences, and one-sided paired t tests elucidated algorithm performance differences between electrode referencing configurations. A Bland-Altman test compared methods to the ground truth consensus across subject-level and reference strategy-level strata, wherein nonsignificant *p*-values, roughly equal to MSE at or smaller than five (median MSE of all algorithms), compared to expert inspection determined acceptable peak localization accuracy. Histograms and UpSet plots^49^ were employed to visualize the range of MBPs and the degree of concordance between expert reviewers and objective algorithms.

### Supplementary information

Supplemental material

## Data Availability

Summary data for peak determinations based on individual reviewers and algorithms will be available at a dedicated OSF site https://osf.io/tuvgc/ .

## Code availability

Code will be made available upon reasonable request to the corresponding author, provided that appropriate data sharing agreements are in place.
