# Supplementary material for: Comparison of beta peak detection algorithms for data-driven deep brain stimulation programming strategies in Parkinson’s disease
Source: NPJ Parkinsons Dis. 2024 Aug 9;10:150. doi: 10.1038/s41531-024-00762-7 (PMC11315991; doi:10.1038/s41531-024-00762-7)
Supplement: Supplementary file 1 — Supplemental material [file 41531_2024_762_MOESM1_ESM.pdf]

Supplemental Information

Supplemental Table 1: Subject Demographics

Subject	Sex (F/M)	Age[1]	Disease Duration[1]	MDS-UPDRS-III OFF/ON*
1	F	61	8	30/15
2	F	56	11	44/21
3	F	55	8	47/33
4	M	67	8	31/9
5	M	62	13	47/39
**MEAN**	**4/6**	**61.4**	**9.20**	
**STD**	**-**	**6.69**	**3.71**	

* "ON" and "OFF" refer to pre-operative medication status, "OFF" was measured after at least 12 hours withhold of medication. Age as in years. F: female, M: male, STD: standard deviation, 1: in years.

Supplemental Table 2: Reviewer KS p-values and Rank-Sum p-values

Reviewer ID	KS P-value	RS P-value	Sign.
1	0.97	0.36	No
2	1.00	0.71	No
3	1.00	0.74	No
4	0.78	0.31	No
5	0.49	0.06	No
6	1.00	0.94	No
7	0.87	0.38	No

Supplemental Table 3: Algorithm KS p-values and Rank-Sum p-values

Reviewer ID	KS P-value	RS P-value	Sign.
I	**1.1E-06**	**5.4E-06**	Yes (both)
II	**0.01**	**2.0E-05**	Yes (both)
III	1.00	0.71	No
IV	0.98	0.65	No
V	0.99	0.92	No
VI	**0.02**	**0.00**	Yes (both)
VII	0.35	0.09	No
VIII	0.81	0.39	No
IX	0.93	0.73	No
X	**0.00**	**0.00**	Yes (both)

Supplemental Figure 1: Expanded Reviewer Upset Plot

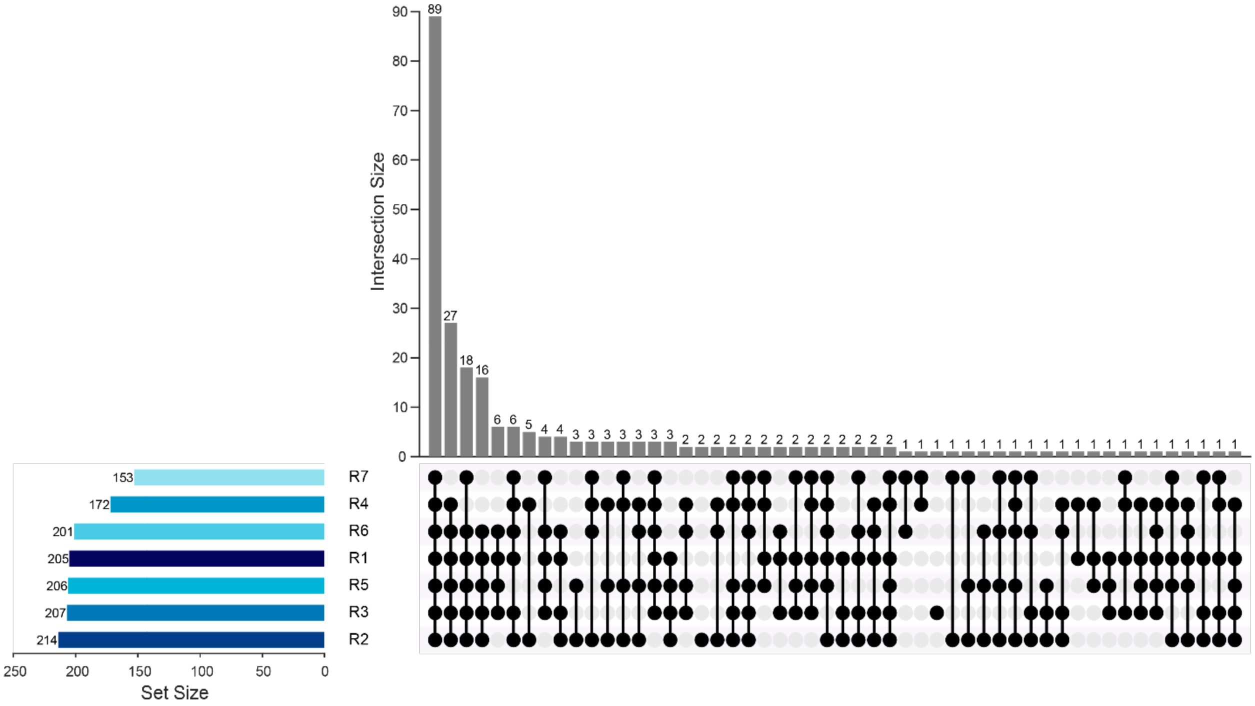

UpSet plot depicts the combination of reviewer groups that accurately identified the consensus peak for a set of PSDs. Horizontal bar chart depicts the total number of PSDs across sets in which the reviewer accurately identified the consensus peak. Vertical bar chart represents the frequency of set intersections (meaning the combination of reviewers agreeing upon a set of PSDs) ordered from the highest number of intersections (most common set combinations) to the lowest number of intersections (least common set combinations). The connected dot plots indicate the reviewers included in each intersection set.

Supplemental Figure 2: Expanded Algorithm Upset Plot

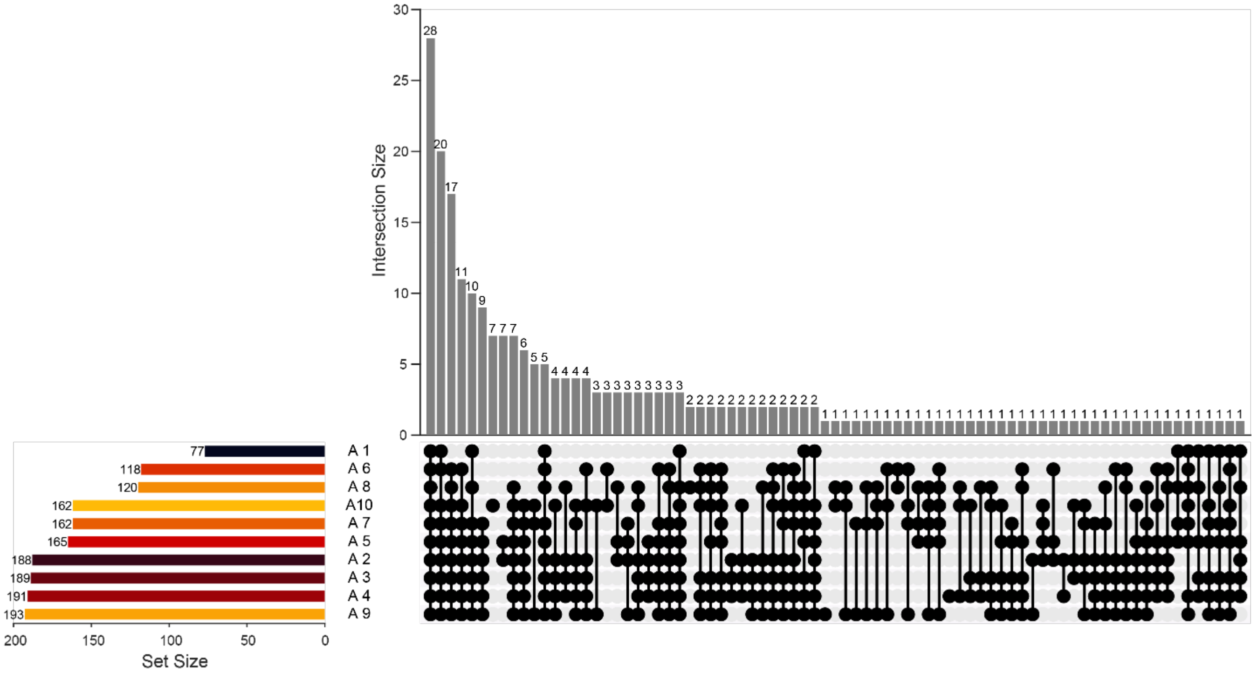

UpSet plot depicts the combination of algorithm groups that accurately identified the consensus peak for a set of PSDs. Horizontal bar chart depicts the total number of PSDs across sets in which the algorithm accurately identified the consensus peak. Vertical bar chart depicts the number of intersections between sets (meaning the combination of algorithms agreeing upon a set of PSDs) ordered from the highest number of intersections (most common set combinations) to the least common. The lower connected dot plots indicate the algorithms included in each intersection set.
